# The Preventive Effect of Ulinastatin on Blood–Brain Barrier Dysfunction in Rats with Postoperative Cognitive Dysfunction After General Anaesthesia with Isoflurane

**DOI:** 10.3390/ijms252312505

**Published:** 2024-11-21

**Authors:** Eun-Hwa Cho, Eun-Hye Seo, Seung-Wan Hong, Seong-Hyop Kim

**Affiliations:** 1 Department of Infection and Immunology, Konkuk University School of Medicine, Seoul 05029, Republic of Korea; eunhwa099@naver.com; 2 BK21 Plus, Department of Cellular and Molecular Medicine, Konkuk University School of Medicine, Seoul 05029, Republic of Korea; gmreo@naver.com; 3 Department of Anesthesiology and Pain Medicine, Konkuk University Medical Center, Konkuk University School of Medicine, Seoul 05030, Republic of Korea; kagura2038@naver.com; 4 Department of Medicine, Institute of Biomedical Science and Technology, Konkuk University School of Medicine, Seoul 05029, Republic of Korea; 5 Department of Medical Education, Konkuk University School of Medicine, Seoul 05030, Republic of Korea

**Keywords:** ulinastatin, blood–brain barrier, tissue inhibitor of matrix metalloproteinase-1, postoperative cognitive dysfunction, isoflurane anaesthesia

## Abstract

This study evaluated the effect of ulinastatin on blood–brain barrier (BBB) dysfunction in rats with postoperative cognitive dysfunction (POCD) following general anaesthesia with isoflurane. Specifically, we examined BBB permeability and the expression of tissue inhibitor of matrix metalloproteinase-1 (TIMP-1). Rats in the ulinastatin group received the drug intraperitoneally (50,000 U/mL), while controls received normal saline (1 mL) administered before general anaesthesia. Isoflurane (1.5% volume) anaesthesia was induced for 2 h. Cognitive function was assessed using the Y-maze test. Two days after anaesthesia, BBB permeability was measured using Evans blue, and TIMP-1 expression was evaluated. Both groups experienced cognitive decline following anaesthesia. However, the ulinastatin group showed a more limited decrease (control group, 64.2 ± 19.3 → 30.2 ± 16.2, *p* = 0.008; ulinastatin group, 70.0 ± 15.7 → 66.5 ± 12.0, *p* = 0.67). The ulinastatin group showed a significantly lower permeability of the BBB (0.034 ± 0.003 µg/g in control group vs. 0.005 ± 0.002 µg/g in ulinastatin group, *p* = 0.0001), and also showed a significantly higher value of TIMP-1 expression (5.81 ± 1.94% in control group vs. 13.97 ± 2.59% in ulinastatin group, *p* = 0.0001). Administration of ulinastatin before general anaesthesia mitigated cognitive decline in rats with POCD, likely through the prevention of BBB dysfunction, as evidenced by the lower BBB permeability and increased TIMP-1 expression.

## 1. Introduction

As life expectancy increases, the demand for medical procedures requiring general anaesthesia in elderly patients is also growing. One complication associated with general anaesthesia in this population is postoperative cognitive dysfunction (POCD) [1]. POCD is characterised by a temporary decline in cognitive function, which can persist for several months, and in some cases, for several years [2]. This condition leads to delayed recovery, extended hospital stays, postponed return to work, and a significant reduction in quality of life [2]. Although its exact mechanism is not fully understood, previous studies have suggested a strong association between POCD and blood–brain barrier (BBB) dysfunction [3].

Ulinastatin, a urinary trypsin inhibitor purified from fresh human urine [4], has both protease-inhibitory and anti-inflammatory properties [5]. Therefore, it has been widely used for the treatment of inflammatory diseases, although its availability is limited to China, India, Japan, and Korea [6]. Duan et al. found that ulinastatin significantly reduced the incidence of POCD, attributing this effect to its anti-inflammatory properties [5,7]. Liu et al. reported that ulinastatin improved BBB permeability in rats with ischemia–reperfusion injury [8]. Zheng et al. found that ulinastatin exerted a neuroprotective effect by reducing zonular occludens-1, thereby providing structural support to tight junction of BBB in meningitis [9]. Based on these findings, ulinastatin is expected to play a similar role in preventing or inhibiting BBB dysfunction associated with POCD, although previous studies have primarily focused on its protective effects in specific diseases, targeting particular pathways [10,11,12]. However, no studies have yet reported on the effect of ulinastatin on the BBB in the context of POCD.

We hypothesised that the preventive effect of ulinastatin on POCD might be linked to its ability to prevent or inhibit BBB dysfunction. We evaluated the impact of ulinastatin on BBB dysfunction by measuring BBB permeability and the expression of tissue inhibitor of matrix metalloproteinase-1 (TIMP-1) in rats with POCD following general anaesthesia with isoflurane.

## 2. Results

In total, 12 rats were enrolled in the study and equally distributed into the two groups. No subject was excluded during the study, including screening.

**Figure 1:**
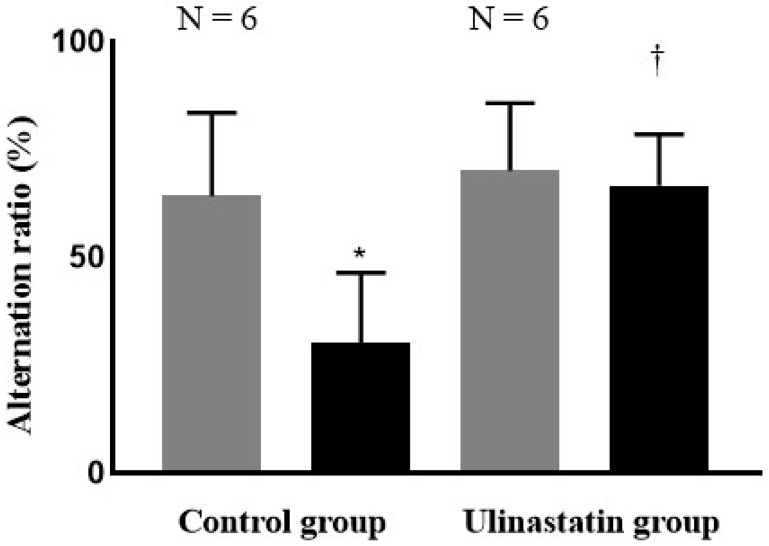
Cognitive function, assessed using Y-maze test, on the day before general anaesthesia (represented with gray bar) and two days after general anaesthesia (represented with black bar). *: *p* = 0.008, compared with on the day before general anaesthesia (represented with gray bar) and two days after general anaesthesia (represented with black bar). ^†^: *p* = 0.001, compared with Control group.

The rates of alternation, which were measured using the Y-maze, were similar between the groups (*p* = 0.58); those of both groups decreased after general anaesthesia, but the decline was less pronounced in the treatment group (control group, 64.2 ± 19.3 → 30.2 ± 16.2, *p* = 0.008; ulinastatin group, 70.0 ± 15.7 → 66.5 ± 12.0, *p* = 0.67) (Figure 1).

**Figure 2:**
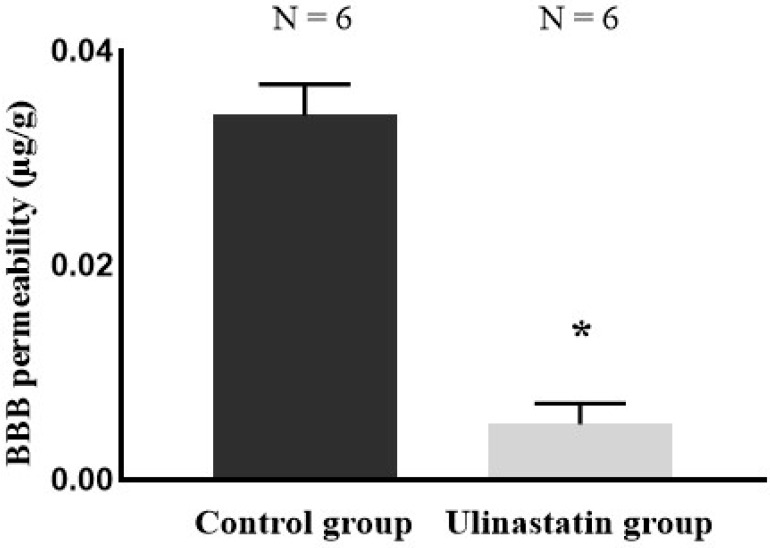
Evans blue staining for blood–brain barrier (BBB) permeability. *: *p* = 0.0001 compared with control group.

The permeability of the BBB significantly differed between the groups, being significantly lower in the treatment group (0.034 ± 0.003 µg/g vs. 0.005 ± 0.002 µg/g; *p* = 0.0001) (Figure 2).

**Figure 3:**
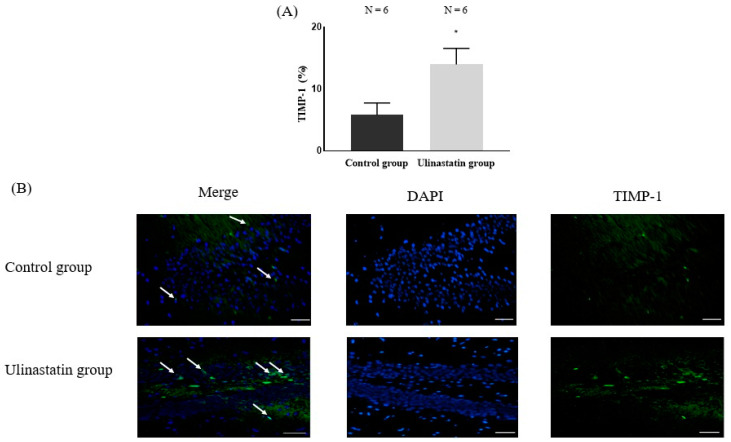
Immunofluorescence staining to detect tissue inhibitor of matrix metalloproteinase-1 (TIMP-1). (**A**) Expression of TIMP-1 and (**B**) Immunofluorescence staining (represented with white arrow) The microscope magnification was taken at 20×. Abbreviations: DAPI, 4′,6-diamidino-2-phenylindole. *: *p* = 0.0001 compared with control group.

The expression of TIMP-1 showed a significant difference between the groups. The ulinastatin group showed a significantly higher value (5.81 ± 1.94% in control group vs. 13.97 ± 2.59% in ulinastatin group, *p* = 0.0001) (Figure 3).

**Figure 4:**
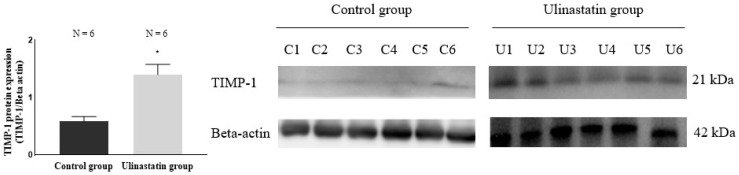
Western blot for quantification of tissue inhibitor of matrix metalloproteinase-1 (TIMP-1) in the brain. Abbreviations: C, control; U, ulinastatin. *: *p* = 0.0001, compared with control group.

Western blot for quantification of TIMP-1 in the brain also showed a significant difference between the groups. The ulinastatin group showed a significantly higher value (0.58 ± 0.09 in control group vs. 1.40 ± 0.18 in ulinastatin group, *p* = 0.0001) (Figure 4).

The ulinastatin group showed significantly lower values of pro-inflammatory cytokines tumor necrosis factor-α (TNF-α) and Interleukin (IL)-1β, and a significantly higher value of the anti-inflammatory cytokine IL-10 (Table 1). However, the anti-inflammatory cytokine IL-4 did not show any difference between the groups (Table 1).

## 3. Discussion

Ulinastatin administration before general anaesthesia prevented a decline in cognitive function, as evaluated by the Y-maze test, in rats with POCD after isoflurane anaesthesia. This protective effect was associated with the inhibition of BBB permeability disruption and a significantly higher expression of TIMP-1. In addition, it was linked to an anti-inflammatory response, as evidenced by the results of the ELISA.

The BBB plays a critical role in maintaining brain homeostasis. Tight junctions between adjacent cells form the core structure of the BBB, limiting paracellular permeability [13]. This allows the BBB to supply nutrients to the brain while shielding it from harmful substances in the blood [14]. When the brain experiences an injury, regardless of cause, neuroinflammation ensues, often leading to BBB dysfunction. The aging process is characterized by a general decline in immune response. The decline is associated with a reduced ability to generate an immune response and increased susceptibility to antigen [15]. It is accompanied by changes in cytokine expression, with increased pro-inflammatory cytokines and decreased anti-inflammatory cytokines [15,16]. Furthermore, a natural decline in BBB function with aging has been reported, even in individuals without any disorders [17]. This suggests that just aging is one of risk factor for POCD. Although the exact mechanism of POCD remains unclear, neuroinflammation and subsequent BBB dysfunction are considered key contributors to its development [3,18]. Under healthy conditions, the permeability of the BBB is tightly regulated, preventing the transport of harmful substances into the brain. However, neuroinflammation can increase BBB permeability, leading to its dysfunction [19]. Cao et al. showed that general anaesthesia with isoflurane changes the morphological structure of the BBB and decreases the expression of tight junction-related proteins, thereby increasing BBB permeability in aged rats [20]. Zhu et al. further demonstrated that POCD is associated with BBB breakdown, which allows the entry of peripheral immune cells into the brain, exacerbating neuroinflammation in mice [21]. Wen et al. reported that restoring BBB permeability and the expression of tight junction-related proteins are protective strategies against POCD in aged mice [22]. Therefore, our finding that ulinastatin administration significantly reduced BBB permeability suggests that the preventive effect of ulinastatin on POCD is strongly associated with its ability to inhibit BBB permeability disruption.

We assessed BBB dysfunction in POCD by measuring BBB permeability and TIMP-1 expression. TIMP-1 is a glycoprotein that plays a critical role in the composition of the extracellular matrix (ECM) [23]. It regulates ECM degradation in vascular walls by inhibiting the activity of matrix metalloproteinases (MMPs) [24], which are responsible for ECM breakdown [25]. The above evidence has shown that TIMP-1 preserves BBB integrity and function, thereby preventing cognitive decline [26,27]. Huang et al. demonstrated that aged mice with POCD showed higher expression of MMP-9, leading to BBB disruption and increased permeability. They also reported that POCD was alleviated in MMP-9 knockout mice, indicating that inhibition of MMP-9 expression protects against POCD [19]. Therefore, TIMP-1 plays a protective role in maintaining BBB integrity [28]. Tang et al. explained that neurological disorders are often associated with BBB breakdown and related microvascular hyperpermeability [29]. They further showed that TIMP-1 provides protection against BBB disruption caused by traumatic brain injury in mice [30]. Saha et al. reported that TIMP-1 improved cognitive behaviour in rodents with Alzheimer’s disease [31]. Li et al. found that MMP-9 expression was increased in mice with cerebral ischemia–reperfusion injury, which was accompanied by a loss of occludin, a tight junction protein at the BBB. They also reported that ulinastatin administration inhibited the increase in MMP-9 and mitigated the loss of occludin, leading to significant improvement in neurological deficits in these mice [32]. Although these findings were not specifically evaluated in the context of POCD, the significant increase in TIMP-1 expression observed in the present study suggests that the preventive effect of ulinastatin on POCD is likely related to the protective role of TIMP-1 in maintaining BBB integrity. Moreover, Zhang et al. demonstrated that ulinastatin administration decreased MMP-9 expression and improved POCD in elderly patients undergoing spinal surgeries, although the study did not directly explore the link between MMP-9 and TIMP-1 [33]. In addition, TIMP-1 was induced and predominantly expressed by astrocytes [34,35]. These findings further support the role of TIMP-1 as an endogenous factor that mitigates MMP activity during neuroinflammation [36].

In this study, the ELISA results further supported the anti-inflammatory effects of ulinastatin, as administration significantly inhibited the release of pro-inflammatory cytokines TNF-α and IL-1β while significantly increasing the release of the anti-inflammatory cytokine IL-10. However, the levels of another anti-inflammatory cytokine, IL-4, did not significantly differ between the groups. Changes in cytokines were also observed in the previous study performed by our group [7]. This may suggest that the effect of ulinastatin on inflammatory cytokines might be selective, although further investigation is warranted.

If the un-anesthetized group had been included in the present study, the findings might be more informative. However, we did not include the un-anesthetized group in the present study. Anaesthesia induces various physiologic changes, including hemodynamic and respiratory alterations, which in turn affect the central nervous system, including the BBB. These physiologic changes should be considered in a comparison between subjects with anaesthesia and those without anaesthesia due to their different physiologic statuses. Actually, the concentration of isoflurane from the experiment might be zero or ignorable on two days after general anaesthesia with isoflurane in the present study. Therefore, the effect of isoflurane on physiologic changes was thought to be limited. However, the effects of isoflurane, regardless of the concentration of isoflurane in the body, on the subjects after general anaesthesia with isoflurane were able to last for a long period of time. POCD may last several months, or even years, after general anaesthesia and surgery, although anaesthetic agents were not detected in the body and the recovery of the body was carried out after surgery. A surgical procedure under anaesthesia should be required in order to establish an exact animal model of POCD. Therefore, the present study might be performed again with three groups: a sham group (just surgical procedure), a control group (surgical procedure under general anaesthesia without ulinastatin), and an intervention group (surgical procedure under general anaesthesia with ulinastatin). The sham group might provide valuable information on the preventive effect of ulinastatin on POCD. However, we decided against including a sham group for several reasons. First, a surgical procedure should be performed using an anaesthetic agent, for example, ketamine, in the sham group due to ethical issues. This meant that it was difficult to isolate the effect of the anaesthetic agent which was used, ketamine, on POCD. Moreover, it was difficult to rule out the effect of the surgical procedure on POCD because the surgical procedure itself induced inflammation. Not including a sham group in the present study did not mean that we neglected the value of baseline comparisons. We just thought that the control group in the present study served as a valid baseline to assess the preventive effect of ulinastatin on POCD.

In the present study, BBB permeability was assessed using Evans blue staining. Evans blue staining is a simple and visually confirmed method. Therefore, the assessment of BBB permeability using Evans blue has been widely carried out. However, it has limitations to its reliability due to non-specific binding, sensitivity issues, and tissue distribution [37]. If recent alternatives such as sodium fluorescein, magnetic resonance imaging, and radiolabeled or fluorescent tracers [38] had been employed in the present study, they might have provided reliable and precise information.

In the present study, immunofluorescence staining from the anterior part of the left hemisphere, as well as Western blotting and ELISA from the posterior part of the left hemisphere, respectively, were performed. Considering the anatomical structure of the hippocampus, the part of the brain responsible for memory and learning, the present study had a limitation in that the analyses in the present study were performed on only a specific portion, not the entire hippocampus, even though this was inevitable because there were not enough samples.

In conclusion, ulinastatin administration prior to general anaesthesia prevented decline in cognitive function in rats with POCD following isoflurane anaesthesia. This protective effect was associated with the prevention or inhibition of BBB dysfunction.

## 4. Materials and Methods

All experiments were conducted in compliance with the National Institutes of Health (NIH) guidelines for the care and use of laboratory animals. After receiving approval from the Institutional Animal Care and Use Committee (IACUC) of Konkuk University (approval number: KU22223–1), all procedures were conducted following IACUC guidelines at the Konkuk University Laboratory Animal Research Center.

### 4.1. Animal Preparation

Eighteen-month-old male Sprague Dawley (SD) rats weighing about 250 g were purchased from Koatech (Pyeongtaek, Korea). The rats were housed in cages with free access to food and water [7,39]. The room was maintained on a 12 h light/dark cycle (lights on at 7:00 and off at 19:00) at a constant temperature of 25 °C [40]. The rats were acclimated to the experimental conditions for 7 days prior to the study and were fed a standard diet ad libitum, with free access to water [7].

### 4.2. Behaviour Test

Following acclimation, the Y-maze test was performed to assess cognitive function both the day before general anaesthesia and 2 days afterward [7]. The Y-maze was constructed in-house [7]. The Y-maze consisted of 3 arms (A, B, and C), each 50 cm long, 25 cm high, and 10 cm wide, arranged at 120° angles to one another [7]. Before the test, all arms were wiped down with 70% alcohol. Arm C was blocked with a board at the centre of the triangular intersection to restrict access [7]. The rat was placed at the end of arm A and allowed to freely explore arms A and B for 15 min to adapt to the maze [7]. After this adaptation period, the rat was removed and rested for 1 h.

After the rest period, the maze was cleaned again with 70% alcohol, and the board blocking arm C was removed to allow free access to all three arms [7]. The rat was placed at the end of arm A, and its movement was recorded for 5 min using a video camera. The total numbers of arm entries and spontaneous alternations were recorded. The alternation ratio was calculated using the following formula: (number of spontaneous alternations)/(total arm entries−2) × 100% [7]. Rats with an alternation ratio < 40% on the day before general anaesthesia were excluded from the study due to preexisting cognitive impairment [7].

### 4.3. Grouping and General Anesthesia

The rats were randomly divided into treatment and control groups, where the former received an intraperitoneal injection of 50,000 U/mL ulinastatin (Ulistin^®^, Hanlim Pharm., Seoul, Republic of Korea) and controls received 1 mL normal saline. After administration, anaesthesia was induced with an intraperitoneal injection of ketamine (100 mg/kg, Yuhan, Seoul, Republic of Korea) and xylazine (10 mg/kg, Sigma-Aldrich, St. Louis, MO, USA). A heating pad was placed on the surgical platform to maintain the rats’ body temperatures at ~37 °C during anaesthesia [7].

The rats were secured in the supine position and fastened to the surgical platform to facilitate endotracheal intubation, for which we used a 16 G catheter 45 mm in length (Dukwoo Medical, Gyeonggi, Republic of Korea) [7]. The correct placement of the catheter was confirmed by observing symmetrical chest expansion [7]. Once the catheter was in place, it was connected to a ventilator (Harvard Apparatus, Holliston, MA, USA). The ventilator settings were as follows: fraction of inspired oxygen (FiO_2_), 0.5; inspired flow rate, 150 mL/min; tidal volume, 6 mL/kg; respiratory rate, 50 breaths/min; inspiration to expiration ratio, 1:1; and positive end-expiratory pressure (PEEP), 5 cm H_2_O [7].

Anaesthesia was maintained with 1.5% isoflurane for 2 h. The ventilator settings were applied throughout the anaesthesia period. After the 2 h anaesthesia period, the isoflurane vaporiser was turned off, and mechanical ventilation was continued until spontaneous breathing fully recovered. Once spontaneous ventilation was confirmed, the 16 G catheter used for endotracheal intubation was removed, and the rat was returned to its cage [7].

### 4.4. Brain Tissue Preparation After Evans Blue Staining for BBB Permeability

Two days after general anaesthesia, following the Y-maze test, the rats were anaesthetised using 5% isoflurane with an oxygen flow of 0.3 L/min and nitrous oxide at 0.7 L/min in an anaesthesia induction chamber. Once the rats lost their ability to right themselves, they were removed from the chamber and placed in a restrainer (Jeungdo Bio & Plant, Seoul, Republic of Korea). After securing the rats in the restrainer, the tails were cleaned with 70% alcohol to prepare for tail vein injection. After confirming blood flow by observing regurgitation from the tail vein, 2 mL/kg Evans blue solution (Sigma, St. Louis, MO, USA) was injected using a 1 mL syringe. Then, the rats were returned to their cages for a 1 h resting period.

Following this rest period, the rats were re-anaesthetised with 5% isoflurane, oxygen at 0.3 L/min, and nitrous oxide at 0.7 L/min in the anaesthesia induction chamber. After confirming successful anaesthesia, the rats were euthanised. The abdominal aorta was exposed through dissection, and 1X phosphate-buffered saline (PBS) was perfused into the abdominal aorta until exsanguination was complete and the liver colour shifted from red to white. Then, the skull was opened, and scissors and forceps were used to extract the brain. A picture of the brain was taken, and the brain was divided into the two hemispheres.

The right hemisphere was used to assess BBB permeability using Evans blue staining and was transferred into a 2 mL Eppendorf tube^®^ (Eppendorf, Hamburg, Germany). The left hemisphere was prepared to analyse TIMP-1 expression via immunofluorescence staining, Western blotting, and inflammatory response analysis using an enzyme-linked immunosorbent assay (ELISA) for cytokine detection. The left hemisphere was evenly divided into two parts, including the hippocampus, using Stainless Steel Brain Matrices (Ted Pella, Riverside, CA, USA) via coronal section: one (anterior) for immunofluorescence staining and the other (posterior) for Western blotting and ELISA. The portion for immunofluorescence staining was stored in a 15 mL conical tube containing 4% paraformaldehyde (PFA; BIOSESANG, Gyeonggi, Korea) at 4 °C, while the portion for Western blotting and ELISA was stored in a microtube at –20 °C.

### 4.5. Quantification of BBB Permeability

After Evans blue staining, the right hemisphere was placed in a Petri dish and kept cool by placing ice underneath. It was weighed, homogenised in 1X PBS at a concentration of 100 mg/mL, and then transferred to a microtube. The microtube was centrifuged at 15,000× *g* for 30 min. Following centrifugation, the supernatant was collected and transferred to a 15 mL conical tube. An equal volume of 50% trichloroacetic acid (Sigma, USA) was added to the microtube in proportion to the amount of 1X PBS used during tissue homogenization. The mixture was vortexed for 2 min and centrifuged again at 15,000× *g* for 30 min. After this second centrifugation, the supernatant was collected and transferred to a 96-well plate. The absorbance of the supernatant was measured using a microplate reader at a wavelength of 610 nm. The absorbance values were used to determine the concentration of Evans blue in the BBB using a standard curve, and were expressed as concentration per gram of brain tissue.

### 4.6. Immunofluorescence Staining for TIMP-1 in the Brain

The left hemisphere, including the dentate gyrus of the hippocampus, which plays key roles in aspects of cognitive process such as memory [41] and exhibits enough TIMP-1 expression compared to other parts [42], after being stored in a 15 mL conical tube, was transferred into a paraffin cassette. The tissue in the paraffin cassette was rinsed with water for 1 h to remove residual 4% PFA. Then, the washed cassette was transferred to a tissue processor (Leica Biosystems, Nussloch, Germany) for paraffin sectioning. The processing program was as follows: 1 h in formalin 1, 1 h in formalin 2, 1 h in 70% alcohol, 1 h in 80% alcohol, 1 h in 90% alcohol, 1 h in 100% alcohol 1, 1 h in 100% alcohol 2, 1 h in xylene 1, 1 h in xylene 2, 2 h in paraffin 1, and 2 h in paraffin 2.

After tissue processing, the paraffin cassette was transferred to a tissue-embedding centre (Leica Biosystems, Germany) for paraffin embedding [7]. Once embedded, the paraffin block was placed in a freezer for 30 min to fix the tissue [7]. After fixation, the tissue was sectioned using a microtome (Leica Biosystems, Germany), and the sections were immersed in a warm water chamber set to 37 °C. After immersion, the tissue sections were transferred onto microscope slides, which were dried on a hot plate at 40 °C for 1 h and then at 56 °C for 30 min [7]. The dried sections were immersed in xylene 1 for 15 min, followed by xylene 2 for 10 min, to remove the paraffin.

After removing the paraffin, the tissue on the microscope slide was rehydrated by sequential immersion in the following solutions: 100% ethanol 1 for 3 min, 100% ethanol 2 for 3 min, 90% ethanol for 3 min, 80% ethanol for 3 min, and 70% ethanol for 3 min [7]. Then, the rehydrated tissue was immersed in 1X citrate buffer for antigen retrieval and microwaved for 5 min three times [7]. After microwaving, the tissue was cooled at room temperature for 20 min and then treated with 5% goat serum as a blocking solution. Following the blocking step, the tissue was washed five times with 1X PBS [7].

The TIMP-1 primary antibody (Bioss, Woburn, MA, USA) was diluted 1:200 in 5% goat serum and applied to the tissue. The slide was incubated at room temperature for 1 h. After incubation, the tissue was washed five times with 1X PBS. The secondary antibody, 488 anti-rabbit antibody (Invitrogen, Waltham, MA, USA), was diluted 1:1000 in 5% goat serum and applied to the tissue, which then was incubated in the dark at room temperature for 1 h. After the secondary antibody incubation, the tissue was again washed five times with 1X PBS.

For nuclear staining, 4′,6-diamidino-2-phenylindole (DAPI; Invitrogen, Waltham, Massachusetts, USA) was diluted 1:2000 in distilled water (DW) and applied to the tissue, which was incubated in the dark at room temperature for 5 min. After incubation with DAPI, the tissue was washed five times with 1X PBS.

Then, the tissue was dehydrated sequentially in 70% ethanol for 3 min, 80% ethanol for 3 min, 90% ethanol for 3 min, 100% ethanol 1 for 3 min, and 100% ethanol 2 for 3 min. This was followed by immersion in xylene 1 for 3 min and xylene 2 for 3 min. After the staining and dehydration process, the tissue was mounted with a cover slip using Antifade Mounting Medium (Vector, Stuttgart, Germany) [7]. The cover slip was sealed with nail polish to prevent drying and movement under the microscope [7]. Then, the stained tissue was observed using a fluorescent microscope (Olympus, Tokyo, Japan) after determination of the temporal pole in the left hemisphere [7].

### 4.7. Western Blotting for Quantification of TIMP-1 in the Brain

The stored left hemisphere, kept in a microtube, was homogenised using PBS and centrifuged at 12,000× *g* for 15 min at 4 °C. After centrifugation, the supernatant was transferred to a new microtube to be used for Western blotting analysis. The protein concentration of the supernatant was quantified using the bicinchoninic acid (BCA) protein assay kit (Thermo, Waltham, MA, USA). A mixture with a total protein amount of 30 μg was prepared by combining the supernatant, 5X sodium dodecyl sulfate-polyacrylamide gel electrophoresis (SDS-PAGE) sample loading buffer (NZYTech, Lisboa, Portugal), and DW in a microtube. The mixture was vortexed and spun down. Then, the microtube containing the mixture was placed in a heat block and heated at 100 °C for 10 min to denature the proteins. After heating, the microtube was cooled on ice for 3 min. Then, the mixture was vortexed again and centrifuged at 12,000× *g* for 5 min at 4 °C.

Next, 1X running buffer (All for Lab, Seoul, Korea) was added to an electrophoresis chamber (Bio-Rad, Hercules, CA, USA), and 10% mini SDS-PAGE gels (Bio-Rad, USA) were placed into the chamber. The prepared mixture was loaded into the gel wells along with the Pageruler^TM^ prestained protein ladder (Thermo, USA). The electrophoresis chamber was sealed with its lid, and the PowerPac HC power supply (Bio-Rad, USA) was used to run the stacking gel at 60 V for 30 min. Afterward, the power supply was increased to 120 V to run the resolving gel for 2 h.

After electrophoresis, cold 1X transfer buffer (All for Lab, Korea) was poured into the chamber, and the separated proteins in the gel were transferred to a membrane. Then, the loaded gels were soaked in 1X transfer buffer (Deajung, Gyeonggi, Korea) to ensure stability. Following the completion of electrophoresis, the proteins in the gel were tagged with specific antibodies, and the electrophoresis chamber was filled with 1X transfer buffer for the transfer process.

The gels tagged with specific antibodies and Immuno-Blot^®^ polyvinylidene difluoride (PVDF) membranes (Bio-Rad, USA) were placed in the transfer buffer. Protein transfer from the gels to the membranes was carried out using the PowerPac HC power supply at 80 V for 150 min. After the transfer, the membranes were washed in 1X Tris-buffered saline with Tween 20 (TBS-T; All for Lab, Korea) in membrane boxes three times for 15 min.

To block nonspecific binding, 5% bovine serum albumin (BSA; Santa Cruz Biotechnology, Dallas, TX, USA) was added to the membrane boxes, and the membranes were incubated at room temperature for 2 h. Following blocking, the membranes were washed again with 1X TBS-T three times for 15 min.

TIMP-1 was used as the primary antibody to evaluate protein expression in the membranes. It was diluted 1:1000 in 5% BSA and incubated with the membranes at room temperature for 1 h. Afterward, the membranes were placed at 4 °C for overnight incubation. The following day, the membranes were washed again with 1X TBS-T three times for 15 min.

For the secondary antibody, goat anti-rabbit immunoglobulin (Ig) G (H+L)-horseradish peroxidase (HRP) (GenDEPOT, Katy, TX, USA) was diluted 1:5000 in 5% BSA and incubated with the membranes at room temperature for 1 h. After incubation, the membranes were washed with 1X TBS-T three times for 15 min.

After washing, the membranes were soaked in Pierce^TM^ ECL Western blotting substrate (ECL; Thermo, USA) and analysed for target protein expression using the iBright CL1000 imaging system (Thermo, USA).

### 4.8. ELISA for Detection of Cytokines

The levels of pro-inflammatory cytokines tumour necrosis factor-α (TNF-α) (Abcam, Cambridge, UK) and interleukin-1β (IL-1β) (Abcam, Waltham, MA, USA), as well as anti-inflammatory cytokines IL-4 and IL-10, were measured using ELISA. ELISA was performed after measurement of the standards of all cytokines to confirm positive controls.

The stored left hemisphere of the brain in the microtube was homogenised using PBS and centrifuged at 12,000× *g* for 15 min at 4 °C. After centrifugation, the supernatant was collected to detect cytokine levels using ELISA kits. The cytokine concentrations were quantified using a microplate reader [7].

### 4.9. Statistical Analysis

The primary outcome of the study was the expression of TIMP-1, while the secondary outcome was the permeability of the BBB, as measured via Evans blue staining. In a pilot study with three rats per group, the expression of TIMP-1 was 7.35 ± 1.16% in controls and 12.46 ± 2.87% in the treatment group. In addition, the permeability values of the BBB were 0.034 ± 0.003 µg/g and 0.005 ± 0.002 µg/g, respectively. Based on these findings, the calculated sample sizes were 12 for the primary outcome and 4 for the secondary outcome, with an α of 0.05 and power of 0.90.

Statistical analyses were performed using GraphPad Prism software version 8.0.1.244 (GraphPad Software, La Jolla, CA, USA). Inter-group statistical significance between the two groups was determined using a *t*-test, while intra-group significance was assessed using an unpaired *t*-test. All data are presented as the number of experiments, mean ± standard deviation, or median (interquartile range). A *p*-value < 0.05 was considered statistically significant.

## Figures and Tables

**Table 1:** Enzyme-linked immunosorbent assay (ELISA) for the detection of cytokines between control group and ulinastatin group.

	Control Group	Ulinastatin Group	*p* Value
Pro-inflammatory cytokines			
TNF-α (pg/mL)	23.62 ± 2.55	6.88 ± 0.90	0.0385
IL-1β (pg/mL)	19.76 ± 5.16	7.70 ± 1.37	0.0002
Anti-inflammatory cytokines			
IL-4 (pg/mL)	23.46 ± 3.74	25.24 ± 3.33	0.4035
IL-10 (pg/mL)	6.66 ± 1.50	15.34 ± 0.95	0.0001

Data are expressed as number of experiments, mean ± standard deviation, or median (interquartile range). Abbreviations: TNF-α, tumour necrosis factor-α; IL, interleukin.

## Data Availability

The datasets are available from the corresponding author upon reasonable request.
